# Long-Term Survival in Octogenarians After Surgical Treatment for Colorectal Cancer: Prevention of Postoperative Complications is Key

**DOI:** 10.1245/s10434-018-6766-1

**Published:** 2018-09-22

**Authors:** Linda B. M. Weerink, Christina M. Gant, Barbara L. van Leeuwen, Geertruida H. de Bock, Ewout A. Kouwenhoven, Ian F. Faneyte

**Affiliations:** 1 Department of Surgery, University of Groningen, University Medical Center Groningen, Groningen, The Netherlands; 2 Department Surgery, Hospital Group Twente, Almelo, The Netherlands; 3 Department of Epidemiology, University of Groningen, University Medical Center Groningen, Groningen, The Netherlands

## Abstract

**Background:**

Whether to treat octogenarians with colorectal cancer (CRC) in the same manner as younger patients remains a challenging issue. The purpose of this study was to analyse postoperative complications and long-term survival in a consecutive cohort of octogenarians who were surgically treated for CRC.

**Methods:**

Octogenarians with primary CRC suitable for curative surgery between January 2008 and December 2011 were included. Data about comorbidities, tumour stage, and complications were retrospectively collected from patient files. Data about survival were retrieved with use of the Dutch database for persons and addresses. To identify factors associated with severe postoperative complications and postoperative survival, logistic regression analyses, and Cox regression analyses were performed. Odds ratios and hazard ratios (HR) with 95% confidence intervals (CI) were estimated.

**Results:**

In a series of 108 octogenarians, median age was 83 years (range 80–94 years). Median follow-up was 47 (range 1–107) months. Major postoperative complications occurred in 25% of the patients. No risk factors for development of severe postoperative complications could be identified. The 30-day mortality was 7%; 1- and 5-year mortality was 19% and 56%, respectively. Overall median survival was 48 months: 66 months in patients without complications versus 13 months in patients with postoperative complications. Postoperative complications were most predictive of decreased survival (HR 3.16; 95% CI 1.79–5.59), even including tumour characteristics, comorbidity, and emergency surgery.

**Conclusions:**

Long-term survival in octogenarians deemed fit for surgery is reasonably good. Prevention of major postoperative complications could further improve clinical outcome.

Colorectal carcinoma (CRC) is the most common malignant disease in elderly people, and the number of elderly patients diagnosed with this disease is expected to increase as the population ages.^1^ Of the 15,549 new CRC patients in 2015 in the Netherlands, 17% were aged 80 years or older.^2^ CRC is, also in the octogenarians, managed with surgical resection of the primary tumour whenever possible.

Octogenarians form a distinctive group of elderly patients, due to the decrease of functional capacity and increase of multimorbidity, impaired cognitive functioning, and disabilities after age 80 years.^3^ How to best treat these increasingly vulnerable octogenarians with CRC remains a challenging issue. For example, elderly patients with rectal cancer less often receive (neo)adjuvant treatment and extended surgical resections and more often undergo surgery with a palliative intent.^4^,^5^ To decide whether to offer older patients the same invasive treatment as younger patients, knowledge about the differences in postoperative outcomes between older and younger patients is important. In literature, a discrepancy between the reported postoperative outcomes in the elderly exists. Some studies show that postoperative complications occur more frequently in the elderly population compared with younger patients.^6^–^9^ Other studies report smaller differences or no differences at all.^10^–^12^ A similar controversy exists regarding the long-term postoperative survival of elderly after surgical treatment for CRC. Some studies show a decreased survival in elderly patients with CRC compared with younger patients.^1^,^9^,^12^–^15^ In contrast, some studies report fairly good long-term survival in the elderly.^12^,^16^–^18^

The purpose of this study was to analyse postoperative complications and long-term survival in a consecutive cohort of octogenarians who were surgically treated for CRC. After comparison of our results with the existing literature, an assessment postoperative complications and impaired long-term survival octogenarians is made.

## Methods

### Study Design

This study is a retrospective cohort study consisting of all assessable octogenarians who were surgically treated between 1 January 2008 and 31 December 2011 for CRC in the Hospital Group Twente (ZGT), a teaching hospital with an adherence area of 300,000 patients in the East of The Netherlands. Treatment was based on the then applicable Dutch national evidence-based guidelines for diagnosis, treatment, and follow-up of colorectal cancer.^19^ Dedicated colorectal surgeons and surgical oncologists performed the surgical procedures. The study protocol was reviewed and approved by the local medical ethical committee.

### Patients

**Fig. 1:**
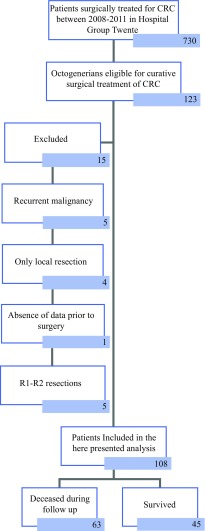
Patient inclusion scheme

All octogenarians with a diagnosis of primary CRC, who were surgically treated with curative intent were included in the analysis. Exclusion criteria were only local resections (e.g., TEM procedure), histological diagnosis not being adenocarcinoma of the colon or rectum, R1 and R2 resections, and the absence of data in follow-up. Patient selection is showed in Fig. 1.

### Data Collection

We retrospectively retrieved the relevant data about patient’s characteristics and data about ASA score, comorbid conditions, and postoperative complications from the medical records. Postoperative complications were graded with use of the Clavien-Dindo classification.^20^ Complications graded III–V on the Clavien-Dindo classification were considered as severe complications. Comorbidity was assessed with use of the Charlson Comorbidity Index (CCI).^21^ A CCI score ≥ 3 was considered as a high burden of comorbidity. To evaluate the risk of malnutrition the Short Nutritional Assessment Questionnaire (SNAQ) was used.^22^ A score of 2 and higher was considered as a high risk of malnutrition. Data about the pT and pN stages and radicality of tumour removal were collected from the pathology reports. To assess whether a patient was still alive, data were retrieved from the Dutch basic registration of persons and addresses (May 2017).

### Endpoints

Primary endpoints were the development of postoperative complications, overall mortality, and survival after primary treatment.

### Statistical Analysis

Baseline characteristics of patients and their disease were described, as well as postoperative complications. To identify variables influencing the survival of elderly, univariate Cox regression analyses were performed. Hazard ratios (HRs) with 95% confidence intervals (CIs) were estimated. To adjust for potential confounders, the baseline variables that significantly (*P* < 0.10) influenced survival were included in the multivariate regression analyses. Kaplan–Meier curves for the analysis of postoperative survival were created. To identify risk factors for developing severe postoperative complications, logistic regression analyses were performed and odds ratios (ORs) with 95% CIs were estimated. *P* value < 0.05 was considered statistically significant. All statistical analyses were performed with SPSS version 23.0 (SPSS Inc., Chicago, IL).

## Results

### Patients

A total of 108 patients were assessed in this study. Most of the patients (80.5%) underwent surgery in an elective setting. The median age was 83 (range 80–94) years; the majority of patients were aged 80–85 years (75.0%). A total of 83 patients (76.9%) were treated for a colon carcinoma and 25 (23.1%) patients were treated for a rectum carcinoma. The majority of the patients were female and did have an ASA II or ASA III classification and a CCI score < 3. A total of 57 patients (44.8%) had a moderate to severe risk of malnutrition. A laparoscopic resection of the tumour was performed in 33 patients (30.6%; Table 1). Most patients presented with a pT3 tumour (53.8%) and no lymph node involvement (65.4%) on pathological evaluation of the resected tissue.

**Table 1:** Baseline characteristics

Variable	Patients (*N* = 108)
Age^a^	83.0 (80–94 years)
Gender	
Male	41 (38.0%)
Female	67 (62.0%)
ASA score	
ASA I	2 (2.0%)
ASA II	48 (47.5%)
ASA III	48 (47.5%)
ASA IV	3 (3.0%)
CCI	
< 3	87 (80.6%)
≥ 3	21 (19.4%)
SNAQ score	
No risk of malnutrition	58 (55.2%)
Risk of malnutrition	57 (44.8%)
Setting of surgery	
Elective	87 (80.6%)
Urgent	21 (19.4%)
Type of surgery	
Open procedure	75 (69.4%)
Laparoscopic procedure	33 (30.6%)
pT stage	
pT 0–1–2	26 (24.5%)
pT 3–4	80 (75.5%)
pN stage	
pN 0	70 (65.4%)
pN 1–2	37 (34.6%)
Postoperative complications	
Anastomotic leakage	10 (9.3%)
Surgical site infection	6 (5.6%)
Pneumonia	8 (7.4%)
Cardiac complications	8 (7.4%)
Thrombo-embolic complications	2 (1.9%)
Neurological complications	1 (0.9%)
Other infectious complications^b^	15 (13.9%)

*ASA* American Society of Anaesthesiologists, *CCI* Charlson Comorbidity Index, *SNAQ* Short Nutritional Assessment Questionnaire

^a^Presented as median (range)

^b^Pneumonia and surgical site infection excluded

Postoperative complications occurred in 53.7% of the patients with 25.1% being severe postoperative complications (Clavien-Dindo score III–V). Anastomotic leakage was the most common postoperative complication, followed by pneumonia and cardiac complications (Table 1). In patients undergoing a colon resection anastomotic leakage was present in seven patients (8.4%) versus three patients (12%) with leakage of a rectal anastomosis. There was no difference in the occurrence of severe postoperative complications in patients undergoing elective or emergency surgery (23.8% vs. 25.3%). Furthermore, there was no difference in occurrence of anastomotic leakage in patients undergoing elective or emergency procedures (9.2% in elective procedures vs. 9.5% in emergency procedures) or any other relevant risk factor for the development of anastomotic leakage. Several possible risk factors for developing postoperative complications, such as age, CCI score, ASA score, and nutritional status, were evaluated. None of these possible risk factors were related to a higher risk of developing postoperative complications (Table 2).

**Table 2:** Factors influencing the development of severe postoperative complications [univariate logistic regression yielding odds ratios (OR) and 95% confidence intervals (CI)]

Variable	OR	95% CI	*P* value
Age	0.99	0.56–1.14	0.893
ASA score			
I–II	1		
III–IV	1.34	0.54–3.30	0.527
Gender			
Male	1		
Female	1.24	0.34–1.92	0.624
SNAQ score			
No risk of malnutrition	1		
Risk of malnutrition	1.91	0.80–4.57	0.145
CCI			
< 3	1		
≥ 3	1.53	0.47–5.02	0.485
Neoadjuvant treatment			
No neoadjuvant treatment	1		
Neoadjuvant treatment	1.85	0.65–5.22	0.248
Setting of surgery			
Elective procedure			
Emergency procedure	1.18	0.41–3.37	0.763
Type of surgery			
Laparoscopic procedure	1		
Open procedure	1.47	0.56–3.88	0.432

*ASA* American Society of Anaesthesiologists, *CCI* Charlson Comorbidity Index, *SNAQ* Short Nutritional Assessment Questionnaire

### Mortality and Survival

**Fig. 2:**
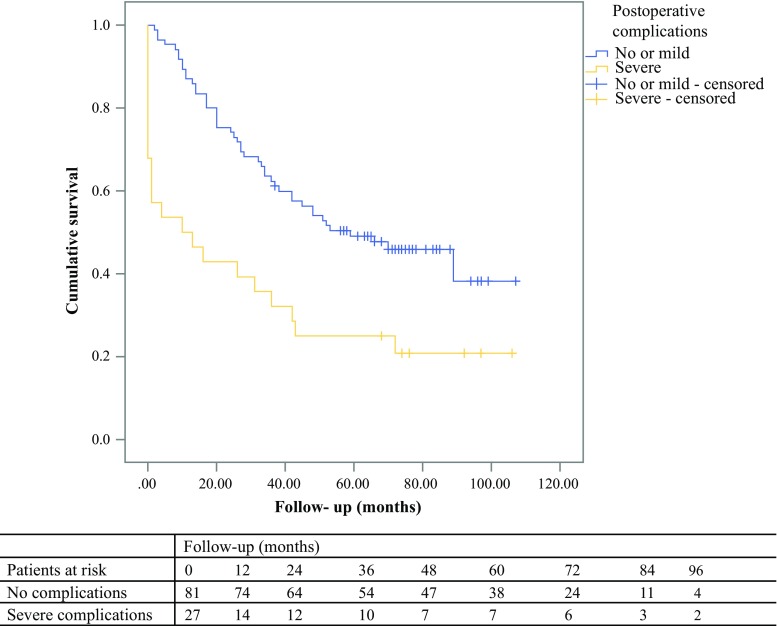
Survival in patients with and without postoperative complications

A total of 63 patients (58.3%) died during follow-up (median follow-up 47 [range 1–107] months). The 30-day mortality was 7.4%, the 1-year mortality was 18.5%, and the 5-year mortality rate was 55.6%. The median survival was 48 months after surgery. In patients with no or mild postoperative complications, the median survival was 66 months, whereas in patients with severe postoperative complications, the median survival was 13 months (Fig. 2). Cause of death was surgery-related in 5 (7.9%), due to CRC and/or metastases in 9 (14.3%), due to other known causes in 13 (20.1%), and unknown in 37 (58.7%) of the deceased patients. In the total population, 4.6% of the patients had a cause of death directly related to surgery.

Multivariate cox regression survival analysis showed that severe postoperative complications (HR 3.16; 95% CI 1.79–5.59), CCI ≥ 3 (HR 2.03; 95% CI 1.15–3.57), emergency surgery (HR 1.86; 95% CI 1.05–3.30), presence of tumour positive lymph nodes in the resected specimen (HR 2.07; 95% CI 1.24–3.45), and age (HR 1.15; 95% CI 1.06–1.25) were independently associated with diminished survival (Table 3).

**Table 3:** Factors influencing the survival in octogenarians with colorectal carcinoma [Cox regression yielding hazard ratios (HR) and 95% confidence intervals (CI)]

Variable	Univariate analysis			Multivariate analysis		
	HR	95% CI	*P* value	HR	95% CI	*P* value
Age	1.12	1.04–1.21	0.005	1.15	1.06–1.25	0.001
Setting of surgery						
Elective surgery	1			1		
Emergency procedure	1.30	1.08–1.56	0.006	1.86	1.05–3.30	0.033
pN stage						
pN0	1			1		
pN1–2	2.28	1.38–3.77	0.001	2.07	1.24–3.45	0.005
Complications						
No or mild postoperative complications C–D 0—I–II	1			1		
Severe postoperative complications C–D III–IV^a^	2.46	1.45–4.17	0.001	3.16	1.79–5.59	< 0.001
CCI						
< 3	1			1		
≥ 3	2.04	1.18–3.53	0.010	2.03	1.15–3.57	0.014
pT stage						
pT 0–1–2	1			1		
pT 3–4	1.88	0.98–3.62	0.058	1.32	0.64–2.71	NS^π^
SNAQ score						
No risk of malnutrition	1					
Risk of malnutrition	1.17	0.72–1.93	0.525			
ASA score						
I–II	1					
III–IV	1.56	0.92–2.66	0.100			
Gender						
Male	1					
Female	1.15	0.69–1.93	0.586			
Type of surgery						
Laparoscopic procedure	1					
Open procedure	1.29	0.74–2.25	0.368			

*ASA* American Society of Anaesthesiologists, *CCI* Charlson Comorbidity Index, *SNAQ* Short Nutritional Assessment Questionnaire

^a^Not statistically significant

## Discussion

With a study population of 108 patients, our study is the largest study focussing on both postoperative complications and long-term survival in octogenarians. Twenty-five percent of the patients developed major postoperative complications. No significant risk factors for the development of postoperative complications were present in our study. Approximately 40% of octogenarians with CRC survived for a period of at least 5 years after surgery. Factors most strongly associated with an impaired survival were the development of postoperative complications, higher burden of comorbidity, emergency procedures, and the presence of tumour-positive lymph nodes. The influence of increased age on the postoperative survival was less striking.

In our study, 25% of the patients developed major postoperative complications. Although high, this is a relatively low percentage compared with the 21–61% complication rate in octogenarians reported in literature.^6^,^7^,^23^ However, it is comparable to studies, including younger patients, reporting a complication rate of 21–35%.^9^–^12^,^24^ This might indicate that the population included in this study was relatively fit before surgery. We found no significant risk factors for the development of postoperative complications. In comparable studies in younger populations, several risk factors, such as age, higher ASA score, and preoperative comorbidity and functioning, has been reported.^6^,^7^,^23^ Research focused on the elderly population showed that risk factors in the elderly differ from risk factors in a younger population and include risk factors, such as increased frailty.^25^,^26^ Even though comorbidity is an aspect of frailty, in our population a higher burden of comorbidity alone did not predict the development of postoperative complications. This might indicate that the integrated concept of frailty is more predictive for the development of complications in this patient group than each aspect of frailty separately. The absence of finding risk factors for the development of postoperative complications in our study can be explained by the fact that the information on other aspects of frailty and factors, such as cognitive functioning and functioning in daily life is very limited.

The 30-day and 1-year survival rates in our study are in accordance with literature, even though these survival rates are diminished compared with the younger population.^27^,^28^ This excess mortality in the elderly has been described before, but its aetiology remains complex with several possible risk factors influencing the short-term survival.^1^,^27^,^28^ The 5-year survival rate in our study was approximately 40% in a population with a median age of 83 years, which is at the high end of the spectrum of the 5-year survival in elderly after surgical treatment for CRC reported in the current literature, which ranges from 23 to 40%.^9^,^12^,^16^–^18^ When comparing our survival rate to the remaining life expectancy of healthy 80-year-old persons in the Netherlands, which is 8.4 years for men and 10.1 years for women, our data indicate that octogenarians can approach a normal life expectancy after surgical treatment for CRC.^29^ Our results showed that postoperative complications, higher burden of comorbidity, emergency procedures, and tumour-positive lymph nodes were the main predictors for reduced survival, which is in line with other studies.^7^,^13^,^17^,^30^–^34^ The effect of age on survival is not clear. Some studies describe an important effect of age on survival, whereas in other studies age was less important.^13^,^18^,^30^,^32^,^35^ In our study, we found no strong effect of age on survival. This might be explained by the way the octogenarians were selected in this study; we selected patients who were deemed fit enough to undergo major surgery by both the surgeon and anaesthesiologist and could be operated on with curative intent. This might have led to the selection of a homogenous group of fit octogenarians.

In our study, the occurrence of complications was the strongest risk factor for reduced survival. Although well-selected octogenarians are not necessarily at a higher risk of postoperative complications compared with younger patients, in case of complications, the median postoperative survival is decreased with 53 months. Furthermore, 8% of the deceased patients died of conditions directly related to the surgical procedure, which is relatively high in this relatively fit group of elderly patients.

This underlines the importance of the prevention of postoperative complications in this age category specifically. Therefore, extra considerations are necessary for preventing postoperative complications, which occur predominantly in the elderly population or have more serious course in this population. A condition that occurs predominantly in the elderly is the development of postoperative delirium.^36^,^37^ During the postoperative period, awareness and measures to prevent postoperative delirium are important, because the occurrence of postoperative delirium can possibly be prevented in a substantial number of the cases.^37^ Strategies for screening and correction of polypharmacy, which are associated with an increased risk of delirium, and preventive measures for postoperative delirium should be an integrated part of the surgical treatment of elderly with CRC.^37^

In our population, patients who underwent open procedures did not have a higher complication risk compared with laparoscopic procedures. A possible explanation could be the patient selection in this study. The literature shows that laparoscopy is safe in the elderly with increased comorbidity and leads to better short-term outcomes with equivalent long-term oncologic outcomes compared with open procedures.^38^–^40^ Therefore, further increase of laparoscopic procedures in the elderly might lead to a decrease in postoperative complications.

An important surgical complication in all patients undergoing surgical resection of CRC is anastomotic leakage, because it influences short-term outcomes but also is related to higher long-term mortality.^24^,^41^ The rate of anastomotic leakage in our population is slightly increased compared with the literature.^42^ The aetiology of anastomotic leakages is complex, and a number of possible risk factors, such as emergency procedures, malnutrition, and increased comorbidity have been described.^42^–^44^ In our population, there were no differences in patients with or without an anastomotic leakage with regard to the presence of malnutrition, percentage of emergency procedures, or burden of comorbidity. Furthermore, surgical techniques to create a tension-free anastomosis are important to prevent anastomotic leakage. To our knowledge, no compromises were made in the resection of the colorectal tumours and tension-free anastomosis were created in our population. An explanation for the relatively high rate of anastomotic leakage might be the increased age of our patient group, because research has shown that age per se is an important risk factor for developing anastomotic leakage, probably due to a combination of risk factors more present in the elderly.^45^ Furthermore, increased age is associated with a higher risk of death after anastomotic leakage.^43^ Prevention of anastomotic leakage could be achieved, partly, by change of surgical treatment strategies in the elderly, e.g., bowel diversion with a colostomy instead of a primary anastomosis. The downside of a colostomy is the relatively high number of early complications.^46^ Even though most complications are relatively minor from a medical point of view, including retraction, leakage, or skin irritation, these complications could greatly diminish quality of life.^46^–^48^ Therefore, patient consultation about the risk of anastomotic leakage and subsequently higher mortality risk versus the possible loss of quality of life after colostomy is important in the decision of whether to perform an anastomosis or colostomy.

The impact of survival in the first postoperative year on the total survival in elderly with CRC has been described in literature; if patients survive the first year after surgery, there is a reasonably good long-term survival.^27^,^49^ Our results show that postoperative complications are a great risk of mortality in the early postoperative period. Therefore, prevention of postoperative complications can contribute to better 1-year and long-term survival. For prevention of postoperative complications in the elderly, the involvement of a geriatrician in perioperative decision-making and care, in addition to tailor-made surgical strategies, can be helpful. A geriatrician, possibly with use of a comprehensive geriatric assessment (CGA), can help to select those patients who might benefit from additional measures in the preoperative period. The CGA, which focuses not only on physical domains but also on domains, such as cognition and functioning, has proven to be useful in the detection of frail patients who are at an increased risk for developing postoperative complications.^25^,^26^ In these frail patients, management of patient and family expectations on outcome before surgery is important and should become more integrated in preoperative decision-making. In the future, the use of CGAs might help to select patients who can benefit from rehabilitation programs, although the exact content and duration of these programs is not clear at present. The geriatric consultation should extend to the postoperative period, because it can improve early detection, treatment, and possibly prevention of postoperative complications.

The strengths of this study are the duration of the follow-up period and the relatively large group of patients included. Furthermore, the survival status of the patients has been checked with the Dutch basic registration of persons and addresses, providing reliable information about the actual survival. With a follow-up period extending to almost 9 years, this study gives insights in the factors that influence the long-term survival.

The main limitation of this study is that only octogenarians who underwent surgical treatment were included, and selection bias seems plausible. The healthier patients were selected for surgical treatment. However, what we can learn from this data is that surgical treatment in octogenarians diagnosed with cancer can have good results.

Furthermore, only ASA score and CCI were used to evaluate the condition of the patients, leading to limited information about the functional status of the patients. Information about functioning in daily life, frailty, muscle strength, cognitive functioning, general performance, and presence of sarcopenia was not recorded. These factors could provide better insight to the condition of the patients and could reveal additional risk factors for development of postoperative complications and impaired survival in octogenarians surgically treated for CRC. In future research, these factors should be considered.

## Conclusions

If an octogenarian is deemed fit enough to undergo colorectal surgery, prevention of major postoperative complications might contribute to improvement of postoperative outcomes. Further research is needed to evaluate which patients are at risk to develop postoperative complications.

## References

[1] Simmonds PD, Best L, George S, Baughan C, Buchanan R, Davis C (2000). Surgery for colorectal cancer in elderly patients: a systematic review. Colorectal Cancer Collaborative Group. Lancet..

[2] Integraal Kankercentrum Nederland. Cijfers over kanker. https://www.cijfersoverkanker.nl/selecties/dataset_1/img5a78b65f40031?type=bar. Updated 2016. Accessed 11 Mar 2016.

[3] Santoni G, Angleman S, Welmer AK, Mangialasche F, Marengoni A, Fratiglioni L (2015). Age-related variation in health status after age 60. PLoS ONE.

[4] Serra-Rexach JA, Jimenez AB, Garcia-Alhambra MA (2012). Differences in the therapeutic approach to colorectal cancer in young and elderly patients. Oncologist..

[5] Chang GJ, Skibber JM, Feig BW, Rodriguez-Bigas M (2007). Are we undertreating rectal cancer in the elderly? An epidemiologic study. Ann Surg..

[6] Turrentine FE, Wang H, Simpson VB, Jones RS (2006). Surgical risk factors, morbidity, and mortality in elderly patients. J Am Coll Surg..

[7] Kim YW, Kim IY (2016). Factors associated with postoperative complications and 1-year mortality after surgery for colorectal cancer in octogenarians and nonagenarians. Clin Interv Aging.

[8] Bircan HY, Koc B, Ozcelik U, Adas G, Karahan S, Demirag A (2014). Are there any differences between age groups regarding colorectal surgery in elderly patients?. BMC Surg..

[9] Hermans E, van Schaik PM, Prins HA, Ernst MF, Dautzenberg PJ, Bosscha K (2010). Outcome of colonic surgery in elderly patients with colon cancer. J Oncol..

[10] Pirrera B, Lucchi A, Gabbianelli C (2017). E.R.A.S. pathway in colorectal surgery in elderly: our experience: a retrospective cohort study. Int J Surg..

[11] Khan MR, Bari H, Zafar SN, Raza SA (2011). Impact of age on outcome after colorectal cancer surgery in the elderly—a developing country perspective. BMC Surg..

[12] Mothes H, Bauschke A, Schuele S, Eigendorff E, Altendorf-Hofmann A, Settmacher U (2017). Surgery for colorectal cancer in elderly patients: how can we improve outcome?. J Cancer Res Clin Oncol..

[13] Tan KY, Kawamura Y, Mizokami K (2009). Colorectal surgery in octogenarian patients—outcomes and predictors of morbidity. Int J Colorectal Dis..

[14] Pedrazzani C, Cerullo G, De Marco G (2009). Impact of age-related comorbidity on results of colorectal cancer surgery. World J Gastroenterol..

[15] Roder D, Karapetis CS, Wattchow D (2016). Colorectal cancer treatment and survival over three decades at four major public hospitals in South Australia: trends by age and in the elderly. Eur J Cancer Care (Engl)..

[16] Schiffmann L, Ozcan S, Schwarz F, Lange J, Prall F, Klar E (2008). Colorectal cancer in the elderly: surgical treatment and long-term survival. Int J Colorectal Dis..

[17] Sheridan J, Walsh P, Kevans D (2014). Determinants of short- and long-term survival from colorectal cancer in very elderly patients. J Geriatr Oncol..

[18] Latkauskas T, Rudinskaite G, Kurtinaitis J (2005). The impact of age on post-operative outcomes of colorectal cancer patients undergoing surgical treatment. BMC Cancer..

[19] Landelijke werkgroep Gastro Intestinale Tumoren. Richtlijn colorectaal carcinoom. Richtlijn colorectaal carcinoom. http://www.oncoline.nl/coloncarcinoom. Updated 2014. Accessed 3 July 2014.

[20] Clavien PA, Barkun J, de Oliveira ML (2009). The clavien-dindo classification of surgical complications: five-year experience. Ann Surg..

[21] Charlson ME, Pompei P, Ales KL, MacKenzie CR (1987). A new method of classifying prognostic comorbidity in longitudinal studies: development and validation. J Chronic Dis..

[22] Kruizenga HM, Seidell JC, de Vet HC, Wierdsma NJ, van Bokhorst-de van der Schueren MA (2005). Development and validation of a hospital screening tool for malnutrition: the short nutritional assessment questionnaire (SNAQ). Clin Nutr..

[23] Manilich E, Vogel JD, Kiran RP, Church JM, Seyidova-Khoshknabi D, Remzi FH (2013). Key factors associated with postoperative complications in patients undergoing colorectal surgery. Dis Colon Rectum..

[24] Law WL, Choi HK, Lee YM, Ho JW, Seto CL (2007). Anastomotic leakage is associated with poor long-term outcome in patients after curative colorectal resection for malignancy. J Gastrointest Surg..

[25] Kristjansson SR, Nesbakken A, Jordhoy MS (2010). Comprehensive geriatric assessment can predict complications in elderly patients after elective surgery for colorectal cancer: a prospective observational cohort study. Crit Rev Oncol Hematol..

[26] Huisman MG, Kok M, de Bock GH, van Leeuwen BL (2017). Delivering tailored surgery to older cancer patients: preoperative geriatric assessment domains and screening tools. A systematic review of systematic reviews. Eur J Surg Oncol..

[27] Dekker JW, van den Broek CB, Bastiaannet E, van de Geest LG, Tollenaar RA, Liefers GJ (2011). Importance of the first postoperative year in the prognosis of elderly colorectal cancer patients. Ann Surg Oncol..

[28] van den Broek CB, Dekker JW, Bastiaannet E (2011). The survival gap between middle-aged and elderly colon cancer patients. Time trends in treatment and survival. Eur J Surg Oncol..

[29] Rijksinstituut voor Volksgezondheid en Milieu. Levensverwachting op oudere leeftijd. http://www.eengezondernederland.nl/Trends_in_de_toekomst/Sterfte_en_levensverwachting/Levensverwachting_op_oudere_leeftijd. Accessed 14 Oct 2016.

[30] Heriot AG, Tekkis PP, Smith JJ (2006). Prediction of postoperative mortality in elderly patients with colorectal cancer. Dis Colon Rectum..

[31] Tan KK, Koh FH, Tan YY, Liu JZ, Sim R (2012). Long-term outcome following surgery for colorectal cancers in octogenarians: a single institution’s experience of 204 patients. J Gastrointest Surg..

[32] Ahmed S, Howel D, Debrah S, NORCCAG (Northern Region Colorectal Cancer Audit Group) (2014). The influence of age on the outcome of treatment of elderly patients with colorectal cancer. J Geriatr Oncol..

[33] van Leeuwen BL, Pahlman L, Gunnarsson U, Sjovall A, Martling A (2008). The effect of age and gender on outcome after treatment for colon carcinoma. A population-based study in the Uppsala and Stockholm region. Crit Rev Oncol Hematol..

[34] Makela JT, Kiviniemi H (2012). Surgical treatment of colorectal cancer in patients aged over 80 years. Int J Colorectal Dis..

[35] Millan M, Merino S, Caro A, Feliu F, Escuder J, Francesch T (2015). Treatment of colorectal cancer in the elderly. World J Gastrointest Oncol..

[36] Scott IA, Hilmer SN, Reeve E (2015). Reducing inappropriate polypharmacy: the process of deprescribing. JAMA Intern Med..

[37] Korc-Grodzicki B, Root JC, Alici Y (2015). Prevention of post-operative delirium in older patients with cancer undergoing surgery. J Geriatr Oncol..

[38] Roscio F, Boni L, Clerici F, Frattini P, Cassinotti E, Scandroglio I (2016). Is laparoscopic surgery really effective for the treatment of colon and rectal cancer in very elderly over 80 years old? A prospective multicentric case-control assessment. Surg Endosc..

[39] Athanasiou CD, Robinson J, Yiasemidou M, Lockwood S, Markides GA (2017). Laparoscopic vs open approach for transverse colon cancer. A systematic review and meta-analysis of short and long term outcomes. Int J Surg..

[40] Zeng WG, Liu MJ, Zhou ZX (2015). Outcome of laparoscopic versus open resection for transverse colon cancer. J Gastrointest Surg..

[41] Turrentine FE, Denlinger CE, Simpson VB (2015). Morbidity, mortality, cost, and survival estimates of gastrointestinal anastomotic leaks. J Am Coll Surg..

[42] Damen N, Spilsbury K, Levitt M (2014). Anastomotic leaks in colorectal surgery. ANZ J Surg..

[43] Bakker IS, Grossmann I, Henneman D, Havenga K, Wiggers T (2014). Risk factors for anastomotic leakage and leak-related mortality after colonic cancer surgery in a nationwide audit. Br J Surg..

[44] Sciuto A, Merola G, De Palma GD (2018). Predictive factors for anastomotic leakage after laparoscopic colorectal surgery. World J Gastroenterol..

[45] Jung SH, Yu CS, Choi PW (2008). Risk factors and oncologic impact of anastomotic leakage after rectal cancer surgery. Dis Colon Rectum..

[46] Formijne Jonkers HA, Draaisma WA, Roskott AM, van Overbeeke AJ, Broeders IA, Consten EC (2012). Early complications after stoma formation: a prospective cohort study in 100 patients with 1-year follow-up. Int J Colorectal Dis..

[47] Kwiatt M, Kawata M (2013). Avoidance and management of stomal complications. Clin Colon Rectal Surg..

[48] Cottam J, Richards K, Hasted A, Blackman A (2007). Results of a nationwide prospective audit of stoma complications within 3 weeks of surgery. Colorectal Dis..

[49] Dekker JW, Gooiker GA, Bastiaannet E (2014). Cause of death the first year after curative colorectal cancer surgery; a prolonged impact of the surgery in elderly colorectal cancer patients. Eur J Surg Oncol..

